# Research and engineering practice on space characteristics of gangue slurry filling

**DOI:** 10.1038/s41598-023-46222-9

**Published:** 2023-12-12

**Authors:** Wenzhe Gu, Baogui Yang, Hao Pan, Tianqi Song

**Affiliations:** 1 China University of Mining & Technology (Beijing), Beijing, 100083 China; 2 China Coal Energy Research Institute Co., Ltd., Xi’an, 710054 Shaanxi China; 3 Xi’an University of Science and Technology, Xi’an, 710054 Shaanxi China

**Keywords:** Civil engineering, Mechanical engineering

## Abstract

The macroscopic characteristics of the fractured space and the microscopic pore features are critical factors determining the effectiveness of gangue slurry backfilling. To identify the key areas for slurry backfilling, a combination of theoretical analysis, simulation experiments, and on-site industrial trials was used to reveal the movement laws of backfilling space overlying the fractured zone. The distribution characteristics of voids available for slurry backfilling within the fractured space were explored, and the interaction between gangue slurry and goaf voids was clarified. A formula for calculating the void ratio in the caved zone was derived, and a model for the distribution of voids in the slurry backfilling space was established. This model identified the main areas where slurry backfilling should be focused, namely the overlapping space between the free accumulation zone and the load-influenced zone. Experimental results demonstrated that the porosity of the collapsed rock mass within the goaf follows a negative logarithmic function along both the dip and strike directions, which was consistent with the theoretical calculations. Through in-situ grouting backfilling experiments on the ground, the feasibility of gangue slurry backfilling in the goaf was verified, and the process of interaction between gangue slurry and goaf voids was elaborated. This process generally involves three stages: initial flow, vertical upwelling, and horizontal diffusion, all of which are closely interconnected. Practical engineering applications of gangue slurry backfilling were carried out in the free accumulation zone and the load-influenced zone at the Huangling No. 2 coal mine. The successful validation of underground gangue slurry backfilling technology demonstrated its feasibility and the correctness of the theoretical approach. This research provides new evidence for environmentally friendly disposal of gangue materials.

## Introduction

The issue of gangue emissions accompanying coal production is one of the key factors currently hindering the green and efficient development of the coal industry^1,2^. According to statistics, the accumulated amount of coal gangue in China has exceeded 6 billion tons, forming over 1700 gangue mountains covering an area of more than 200,000 acres, with an annual emission rate increasing to approximately 700 million tons^3^. The surface accumulation of gangue poses a significant threat to the ecological environment of mining areas, contradicting the concept of ecological civilization that values “green mountains and clear water as invaluable assets”^4,5^.

To address the environmental issues and safety hazards caused by surface accumulation of gangue, research on comprehensive utilization and backfilling mining of coal gangue has been conducted both domestically and internationally, and corresponding achievements have been made^6,7^. However, the large volume of gangue and the relatively low comprehensive utilization rate^8–10^, as well as the high cost and limitations of traditional backfilling mining techniques, such as the mutual influence of mining and backfilling processes and the impact of backfilling on working face productivity^11–13^, make existing gangue utilization methods and approaches inadequate for efficient, green, and large-scale disposal of coal gangue. Based on this, some scholars have integrated the advantages and disadvantages of traditional solid backfilling, paste backfilling, and high-water backfilling techniques^14–16^. They proposed a new type of coal gangue slurry backfilling technology by combining caving zone grouting technology, long-distance pipeline transportation technology, and yellow mud grouting technology^17–19^. Coal gangue slurry backfilling technology utilizes the voids formed by the caving and fragmentation of overlying rocks as storage space for gangue, achieving in-situ, green, and harmless disposal of gangue. The filling capacity and efficiency of the slurry are closely related to the structural characteristics and void distribution of the backfilling space within the caving zone, referred to as the slurry backfilling space.

Due to different backfilling purposes, slurry backfilling differs from paste backfilling. Instead of pursuing high filling body strength, slurry backfilling primarily increases the amount of gangue filling by optimizing the slurry mix ratio and process^20–22^. At the same time, to reduce filling costs, the addition of binders is eliminated, but this leads to a decrease in the fine particle content of the matrix, resulting in significant differences in transport and flow performance between coal gangue slurry and traditional paste^23–25^. On the other hand, to achieve a large-scale increase in the amount of gangue filling, the gangue slurry needs to meet the requirements of good flowability within the goaf while satisfying the demand for long-distance transportation. However, research and application of coal gangue slurry backfilling technology are still in the early stages, and the relationship between the distribution characteristics of voids in the caving zone, the key areas for slurry backfilling, and the slurry diffusion characteristics has not been clearly defined.

Therefore, this study comprehensively adopts a combination of theoretical analysis, similarity simulation, and on-site practice to explore the structural characteristics and evolutionary laws of the backfilling space overlying the fractured zone. It aims to clarify the distribution of voids in the slurry backfilling space, understand the interaction between gangue slurry and goaf voids, and validate the findings through on-site engineering practice.

## Theoretical analysis of slurry diffusion in collapse zone

### Theoretical calculation of void distribution in collapse zone

The goaf caving zone possesses irregularity, fragmentation, and differences in compactness, making it an ideal space for grouting and backfilling^26,27^. By establishing a spatial model of the goaf caving zone, the target space for adjacent grouting and backfilling underground mainly concentrates in the area comprising the basic roof, segmental coal pillars, and coal floor.

After coal seam extraction, a trapezoidal-shaped goaf space is formed in the mining area, disrupting the original stress equilibrium and distribution of the surrounding rock. Influenced by secondary stresses, the surrounding rock undergoes deformation, fractures, and collapse^28–30^. The “block-beam” model of the overlying rock structure divides the deformation, movement, and damage of the overlying rock layer vertically into bending zones, fracture zones, and collapse zones, and along the direction, it is divided into coal pillar support zones, detachment zones, and compaction-stabilized zones. Based on the failure characteristics and accumulation status of the caving rocks, the collapse zone along the goaf direction is further divided into natural accumulation zones, load-influenced zones, and re-compaction zones.

The overlying rock in the mining area is a well-organized stack of multiple layers with different thicknesses and strengths. During the movement of the basic roof layer, the inconsistency in deformation occurs due to varying strength and stress, leading to detachment and formation of detachment cracks in the vertical direction. On the other hand, the immediate roof layer directly collapses, accumulates, and leaves voids as the working face advances. Assuming that the overlying rock strata will subside, fracture, sink, or bend under the influence of upper loads and their own gravity, the rock strata, after breaking, will form a stable “brick-like beam” structure. Once the rock strata's sinking is mostly stabilized, the dip curve of the overlying rock resembles the dip curve of the overlying rock in the strike direction.

According to the mechanical model theory of the "block-beam" structure, after the rock layer fractures, a stable “block-beam” structure is formed, and the vertical displacement *w*_*x*_ along the coal seam direction can be fitted by a curve.

1 $$  \left\{ {\begin{array}{*{20}l}    {\omega _{x}  = \omega \left\{ {1 - \left[ {1 + \exp \left( {\frac{{l_{x}  - \left| {l_{x}  - 2x} \right|}}{{0.5l_{i} }} - 2} \right)} \right]^{{ - 1}} } \right\}} \hfill  \\    {\omega  = M - \sum {h_{i} } \left( {K_{{_{i} }}  - 1} \right)} \hfill  \\    {l_{i}  = h_{i} \sqrt {\sigma _{i} /(3q_{i} )} } \hfill  \\   \end{array} } \right. $$

where *ω*_*t*_ represents the subsidence amount of the key layer during the fracture stage as a function of time, *M* is the thickness of the coal seam, Σ*h*_*i*_ is the distance from the rock layer to the roof of the coal seam, *K*_*i*_ is the residual fragmentation coefficient of the rock within Σ*h*_*i*_, *l*_*x*_ is the length along the goaf direction, and *l*_*i*_ is the rock fracture length of the rock layer. Additionally, hi and *σ*_*i*_ are the thickness and tensile strength of the rock layer, respectively, and *q* represents the sum of the self-weight and the upper load of the rock layer.

The length *l*_*x*_ along the goaf direction can be expressed by the following equation:

2 $$ l_{x} = \int_{0}^{T} v (t){\text{d}}t $$

where *T* represents the time from the start of coal mining at the cutting eye to the cessation line, and *v*(*t*) is the mining velocity.

At the same time, in the coal seam dip direction, the subsidence of the "block-beam" also follows a similar curve. Thus, the vertical displacement *w*_*y*_ along the coal seam dip direction can be fitted by the following equation:

3 $$  \omega _{y}  = \omega _{t} \left\{ {1 - \left[ {1 + \exp \left( {\frac{{l_{y}  - 2|y|}}{{0.5l}} - 2} \right)} \right]^{{ - 1}} } \right\}$$

where *l*_*y*_ represents the dip width of the goaf. Assuming that the subsidence of the key layer due to overlying rock failure is simultaneously influenced proportionally by *w*_*y*_, the vertical displacement of the rock layer forming the stable “block-beam” structure can be expressed as follows:

4 $$  \omega (x,y,t) = \frac{{\omega \left\{ {1 - \left[ {1 + \exp \left( {\frac{{\int_{0}^{t} v (t){\text{d}}t - \left| {\int_{0}^{t} v (t){\text{d}}t - 2x} \right|}}{{0.5l_{i} }} - 2} \right)} \right]^{{ - 1}} } \right\} \cdot \left\{ {1 - \left[ {1 + \exp \left( {\frac{{l_{y}  - 2|y|}}{{0.5l_{i} }} - 2} \right)} \right]^{{ - 1}} } \right\}}}{{1 - \left[ {1 + \exp \left( {\frac{{l_{y} }}{{0.5l_{i} }} - 2} \right)} \right]^{{ - 1}} }}  $$

The porosity of fractured rock mass can be expressed as the ratio of the volume of voids between rock fragments in the broken state to the total volume, while its fragmentation coefficient is the ratio of the volume of the rock mass after fragmentation to the volume before fragmentation. The void ratio of the collapse zone is primarily determined by the subsidence amount of the basic roof:

5 $$  \Psi (x,y,t) = 1 - \frac{{h_{z} }}{{h_{z}  + M - \omega (x,y,t)}},x \in \left[ {0,l_{x} } \right],y \in \left[ { - \frac{{l_{y} }}{2},\frac{{l_{y} }}{2}} \right] $$

where *Ψ* represents the void ratio of the collapse zone, *h*_*z*_ is the distance between the basic roof and the coal seam roof.

### Model of void distribution characteristics in slurry filling space

The 301 working face of Huangling No. 2 Coal Mine has been selected as the planned backfilling face. The coal seam at this working face has an average thickness of 4.7 m. The mining length of the working face is 3709 m, and the dip length is 300.5 m. The immediate roof consists of siltstone with an average thickness of 18 m. The roof management method adopts the fully caving method, with a medium-hard roof and a residual compaction factor of 1.1. The measured periodic weighting interval at the site is approximately 25 m, and the basic roof breakage angle is about 65 degrees. Based on comprehensive empirical formulas, measured data, and analysis of the working face roof conditions, the height of the caving zone formed after coal seam extraction at the 301 working face is estimated to be between 9.2 to 16.9 m. Using Eq. (5), the void ratio of the caving zone is calculated.

**Figure 1:**
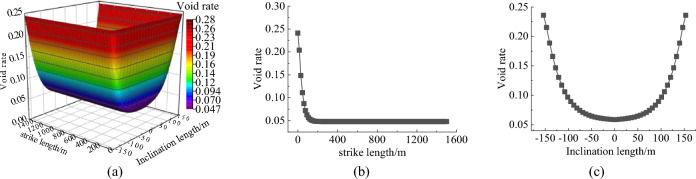
Distribution characteristics of porosity in caving zone.

As shown in Fig. 1, the *x* and *y* axes represent the strike length and dip length of the caving zone, respectively. The void ratio along both the strike and dip directions of the caving zone shows a roughly symmetrical distribution, indicating a clear zoning phenomenon. The central area of the caving zone exhibits a lower void ratio due to recompaction, which gradually increases towards the periphery. The void ratio of the caving zone decreases from the coal wall towards the goaf interior following a negative logarithmic function. The void ratio distribution characteristics depicted in Fig. 1 are crucial for determining the key areas for gangue slurry backfilling and devising appropriate backfilling strategies. These findings provide valuable insights for the application of coal gangue slurry backfilling technology at the 301 working face. The “shell” structure of overlying rock is a typical feature of slurry filling space. The upper control rock layer of the “shell” is the upper boundary of the slurry filling space. The height of the control rock layer determines the height of the slurry filling space. Its structural characteristics reflect the hinge characteristics of the lower broken rock mass. The maximum void ratio is 0.24.

According to the development characteristics of the rock strata controlled by the slurry filling, the slurry filling space can be dip and strike zoned, and a two-dimensional zoning model of the slurry filling space can be established.

The “shell” structure of overlying rock is a typical feature of slurry filling space. The upper control rock layer of the “shell” is the upper boundary of the slurry filling space. The height of the control rock layer determines the height of the slurry filling space. Its structural characteristics reflect the hinge characteristics of the lower broken rock mass. According to the development characteristics of the rock strata controlled by the slurry filling, the slurry filling space can be dip and strike zoned, and a two-dimensional zoning model of the slurry filling space can be established.

The slurry filling space along the strike shows periodic characteristics with the mining of the working face, and is divided into free accumulation area, load affected area and compaction area according to the distribution characteristics.

1. Free accumulation area: the rock block is located in the unloading area of the goaf caving space and is not subject to the action of the rock stratum controlled by the slurry filling. It is in a free accumulation state. The free accumulation area moves forward periodically with the advance of the working face, and the degree of rock fragmentation is high, which can provide a lot of space for slurry filling;
2. Load influence area: the force of roof on collapsed rock mass in the area increases gradually, and the degree of void fragmentation between collapsed rock masses decreases gradually. The area moves forward periodically with the advance of the working face, which can be used as the space for filling slurry;
3. Compaction area: the slurry filling controls the maximum subsidence of rock stratum, and the compaction degree of collapsed rock mass basically reaches a stable state. This area cannot be used as the slurry filling area.

**Figure 2:**
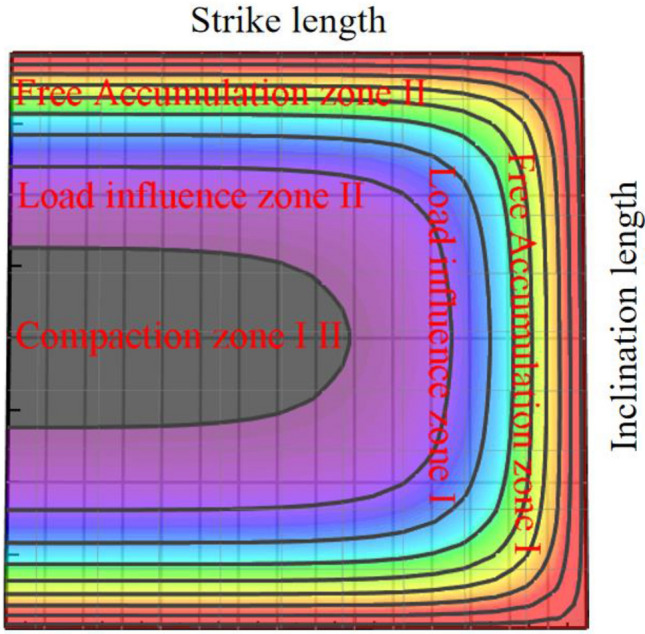
Model of void distribution characteristics in slurry filling space.

Combined with the relationship between the spatial position and the porosity, and considering that the slurry filling does not occur in an independent horizontal or vertical direction, but is the interaction of multiple directions. Based on this, the strike and dip distribution characteristics of the space voids filled with coal gangue slurry are drawn, as shown in Fig. 2. The color depth in Fig. 2 indicates the size of the space gap filled by the slurry. According to the space gap distribution characteristics of slurry filling, the filling position of coal gangue slurry is obtained. This model identified the main areas where slurry backfilling should be focused, namely the overlapping space between the free accumulation zone and the load-influenced zone.

## Characteristics of overburden structure in slurry filling space

### Similar model construction

**Figure 3:**
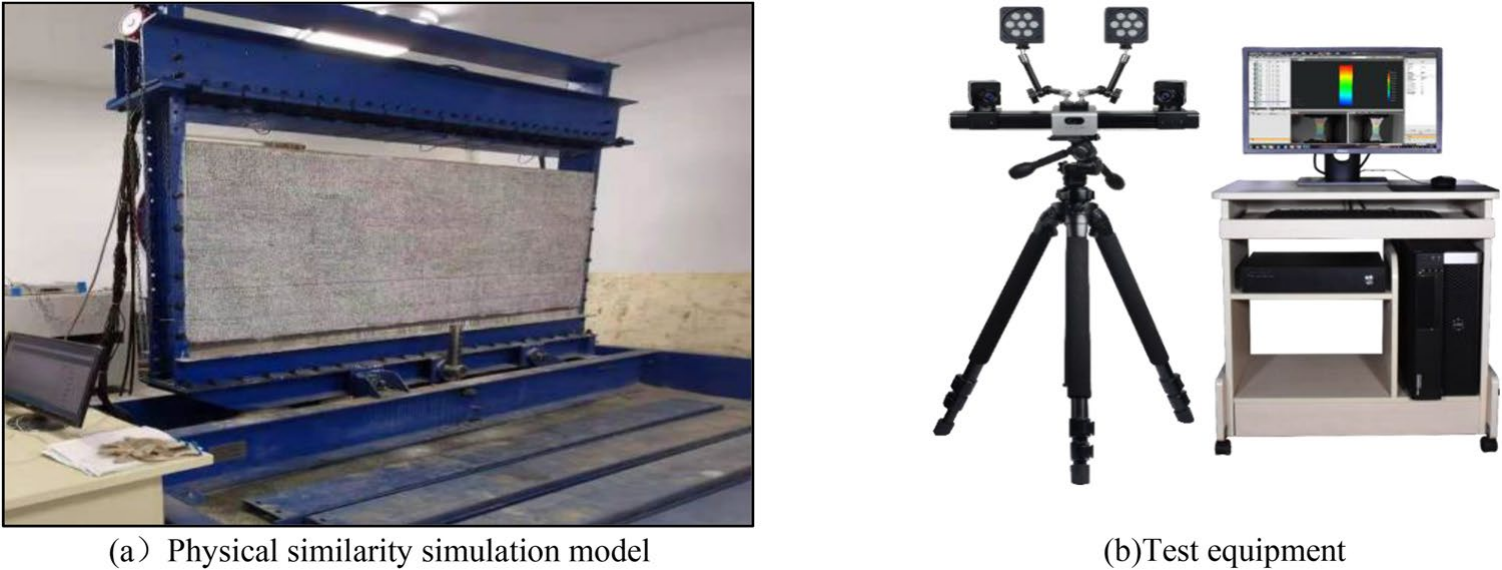
Test System.

In order to explore the structural characteristics and evolution law of the overlying rock in the slurry filling space, establish a physical similarity model based on the geological background of the 301 working face of Huangling No. 2 coal mine. Physical similarity simulation model is shown in Fig. 3. The physical similarity simulation experiment size: length × wide × height = 3.0 m × 0.2 m × 1.20 m, geometric similarity ratio 1:200, the unit weight similarity ratio is 1:1.56, the time similarity ratio is 1:12.25, and the stress similarity ratio is 1:234. The model is excavated from left to right, 3.0 cm each time, and the excavation length is 240 cm. At the same time, in order to reduce the influence of boundary effect on the simulation results, 30 cm boundary protection coal pillars are set on the left and right sides of the model. During the excavation of the model, XTDIC three-dimensional strain measurement system is used to monitor and analyze the surface morphology, displacement and strain of the model.

### Overburden evolution law of slurry filling space

**Figure 4:**
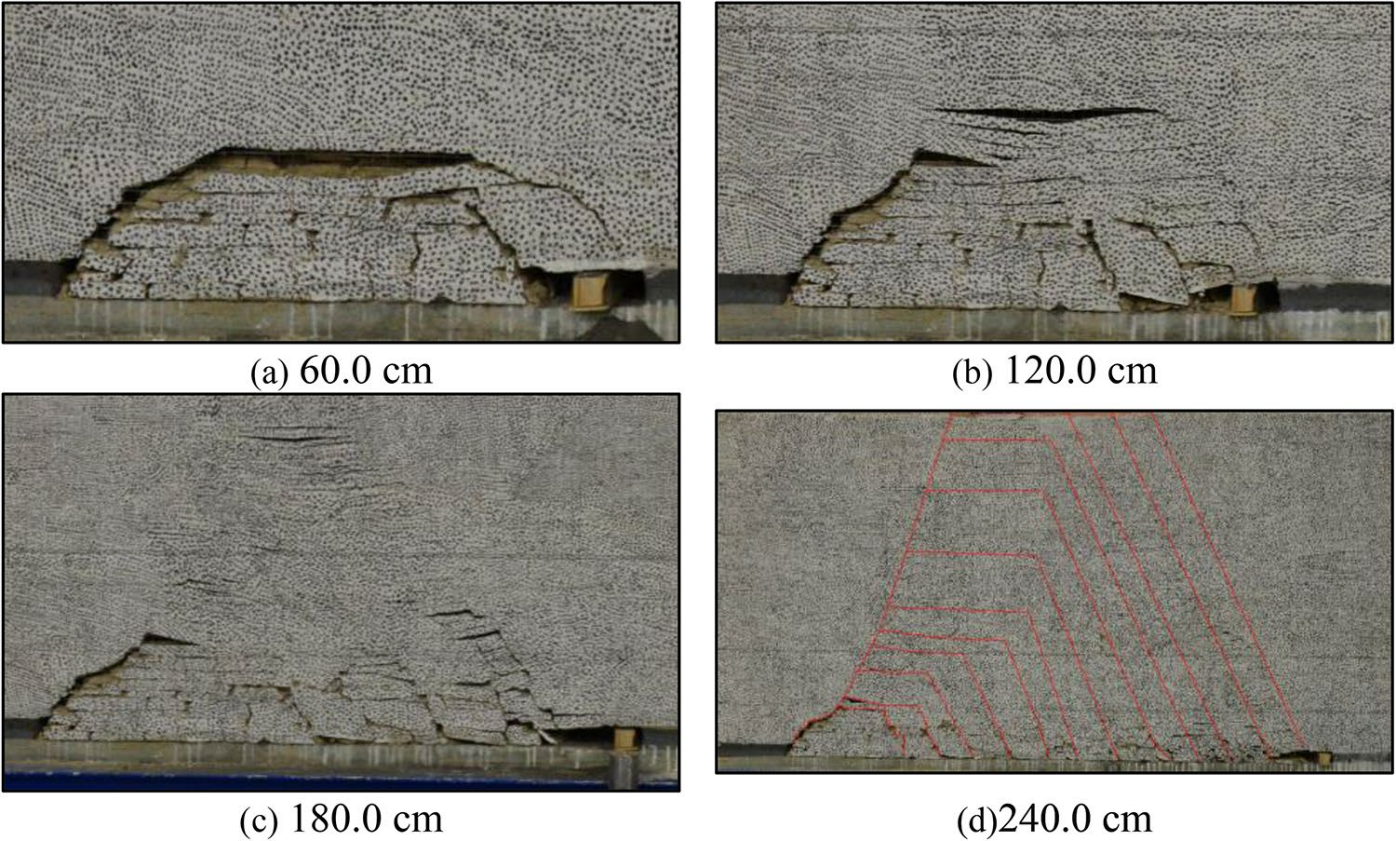
Development process of overburden spatial structure.

The structural evolution characteristics of overburden during model excavation are shown in Fig. 4. Its main characteristics are as follows:

1. Initial Collapse: When the working face advances 60.0 cm, the key rock stratum experiences its first cycle of basic roof collapse, forming a distinct caving “arch” structure. The collapsed roof has a collapse step distance of 13.3 cm, a collapse height of 16.3 cm, and a maximum separation distance of 4.5 cm. Microcracks appear in the overlying rock stratum, and the rock mass in the arch area undergoes significant fragmentation, creating numerous voids that can be utilized as space for filling with coal gangue slurry, as shown in Fig. 4a.
2. Transition to “Beam” Structure: As the working face advances to 120.0 cm, the key rock stratum undergoes a transformation from the “arch” structure to the “beam” structure. The rock mass below the “beam” experiences considerable fracturing, and the void shape becomes evident, meeting the conditions for filling with coal gangue slurry, as shown in Fig. 4b.
3. Strengthening of Fractures and “Shell” Structure: When the working face advances to 180.0 cm, the fractures in the “beam” structure intensify, primarily evolving from horizontal separation fractures to vertical through fractures. The presence of the key rock stratum in the upper part of the “beam” influences the development of interspaces within the overburden fracture space. Fracture development is weaker in the upper part, while there are larger interspaces in the lower part. The key rock stratum, along with the interlayer rock stratum of the collapse arch, forms the “shell” structure of the overburden crushing space, as shown in Fig. 4c.
4. Further Periodic Evolution: With the continuous advancement of the working face, the structural evolution of the key rock stratum exhibits further periodic changes. As the working face continues to advance, the previously described “arch-beam-shell” structure will repeat and form multiple cycles. The height of each structure remains relatively stable within a certain range of excavation height, indicating that the structural evolution characteristics of the key rock stratum are relatively consistent, as shown in Fig. 4d.

The study of the structural evolution characteristics of the key rock stratum also provides reference for the application of gangue slurry filling technology. Due to the presence of numerous voids in the key rock stratum, these voids can be utilized as filling space for coal gangue slurry. Gangue slurry filling technology can effectively fill voids during mining, improve ore recovery, and reduce environmental impacts.

### Space void distribution law of slurry filling

1. Vertical distribution law of porosity in broken rock mass
  The key of slurry filling is to make rational use of the free accumulation area and load affected area to achieve efficient filling of gangue slurry. The Void rate of broken rock mass with different heights in the vertical range of goaf is not a constant, but it decreases from the bottom boundary of collapse zone to the lower boundary of filling control rock layer, and meets the attenuation law of logarithmic function, as shown in Fig. 5. The broken rock mass expansion coefficient within the vertical range of the goaf that varies with the height is fitted according to the following formula:
  6 $$ K_{h} = K_{0} - \lambda \ln (h + h_{0} )(0 \le h \le H_{z} ) $$

**Figure 5:**
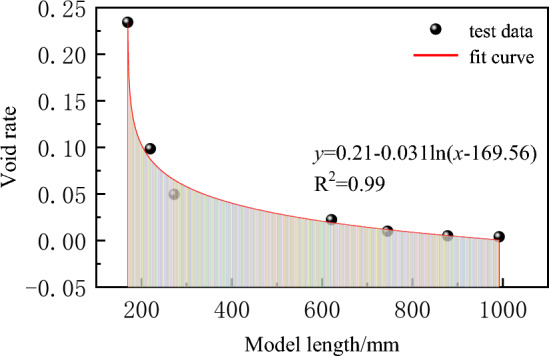
Fitting function of vertical dilatancy coefficient.
  In the formula, *K*_*h*_ is the Void rate of the broken rock mass with the height h from the bottom of the collapse zone; *K*_0_ is the Void rate of the broken rock mass at the bottom of the collapse zone; *λ* Is the attenuation coefficient; *H* is the height of the lower boundary of the rock stratum controlled by slurry filling.
  By fitting the data, the fitting formula of the vertical distribution of the Void rate of broken rock mass is R^2^ = 0.99.
2. Horizontal distribution law of porosity in broken rock mass.
  The broken rock mass expansion coefficient at different positions within the horizontal range of the goaf tends to decrease from the open cut or the working face side up to the depth of the goaf, and meets the attenuation law of the logarithmic function, as shown in Fig. 6. The broken rock mass expansion coefficient within the horizontal range of the goaf that varies with the height is fitted according to the following formula:
  7 $$  K_{{\text{x}}}  = K_{{x_{0} }}  - \lambda \ln \left( {x - x_{0} } \right)\quad \left( {x_{0}  \le x \le L/2} \right) $$
  where *K*_*x*0_ is the coefficient of crushing expansion of rock mass after fracture at *x*_0_ from the mining boundary; *K*_*x*_ is the coefficient of crushing expansion of rock mass after fracture at *x* from the mining boundary; *λ* Is the attenuation coefficient; *L* is the strike or dip distance.

**Figure 6:**
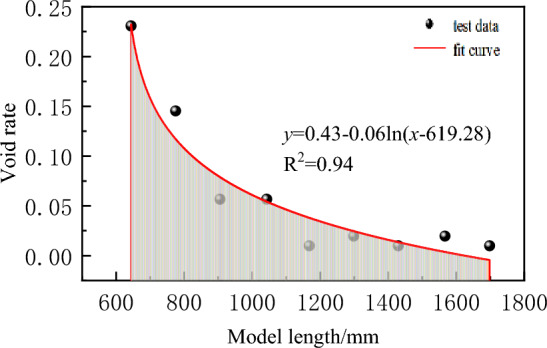
Fitting function of horizontal dilatancy coefficient.

By fitting the data, the fitting formula of the horizontal distribution of the Void rate of broken rock mass is R^2^ = 0.94.

Different from the traditional caving zone, the slurry filling space takes the control rock stratum as the upper boundary, including the traditional caving zone height and some lower fracture zones.

1. The vertical range of slurry filling is larger than the height of the collapse zone, and it is composed of the fracture development area in the lower fracture zone and the collapse zone;
2. The rock stratum controlled by slurry filling is the upper boundary of the slurry filling space. The vertical fractures of the overlying rock stratum are poorly developed, and the lower crushing space is developed, and there is an obvious process of fracture development and closure;
3. The slurry filling space is the evolution process from the void space formed by the broken rock mass to the fracture space formed by the hinge after the complete rock mass is broken, and the void space is the main position of the slurry filling.

The dip direction is affected by the rock stratum controlled by the slurry filling. The space of the broken rock mass under it for slurry filling is mainly divided into three structural forms, which act on five areas, including free accumulation area, load affected area and compaction area.

1. Free accumulation area: it is located in the structural range of arc triangle area near the coal pillar. The rock mass in the area is highly fragmented and is not affected by the load of basic roof and overlying rock, which can provide a lot of space for slurry filling;
2. Load affected area: the slurry filling in the area controls the increase of the subsidence of the rock stratum from the coal pillar to the middle of the working face, which is the main area for slurry flow and diffusion;
3. Compaction area: the slurry filling controls the maximum subsidence of the rock stratum, and the compaction degree of the broken rock mass is relatively high. The slurry cannot be effectively diffused within this range and cannot be used as the slurry filling area.

The grouting filling area determined based on the porosity obtained from experimental testing is in good agreement with the grouting filling area obtained from the theoretical analysis in section “Theoretical analysis of slurry diffusion in collapse zone”, validating the significance of the theoretical analysis.

After the mining of the working face, the overlying strata in the goaf behind the working face collapse. Initially, the basic roof experiences “O–X” fracture due to the first compression. After periodic fracture of the basic roof, the rock blocks along the direction of the working face form a block-beam structure. At the end of the working face, there is a fracture forming an arc-shaped triangular block, and a rock layer supporting structure is formed within the goaf. This structure can bear most of the load of the overlying strata, causing the lower part of the arc-shaped triangular block to collapse and accumulate in a loose state, resulting in larger gaps between the rock blocks, making it an ideal space for slurry filling and utilization. However, in the middle part of the goaf along the dip direction, the rock blocks in the collapse zone bear most of the load of the overlying strata, leading to the collapse zone being densely compacted. The gaps between the rock blocks are smaller, making it difficult to inject slurry, which makes it challenging to fill and utilize this space with slurry.

The study area of this experiment is the lower collapse zone of the arc-shaped triangular zone in the goaf behind the working face (the overlapping space between the free accumulation zone and the load-influenced zone). Due to the presence of thick sandstone at the basic roof and thick medium-grained sandstone at the immediate roof of the coal mine, the hanging effect of the gob-side roof in the goaf is evident after the mining of the working face, and the immediate roof inside the gob-side roadway cannot collapse sufficiently, resulting in a relatively low accumulation height of collapsed rock blocks in this region. In the roof compaction area outside the hanging range of the cantilever, the collapsed rock blocks have a more sufficient accumulation height. As a result, the lower collapse zone of the arc-shaped triangular area exhibits a step-like pattern of collapsed rock accumulation.

## Relationship between gangue slurry and goaf void

### Establishment of field test model

**Figure 7:**
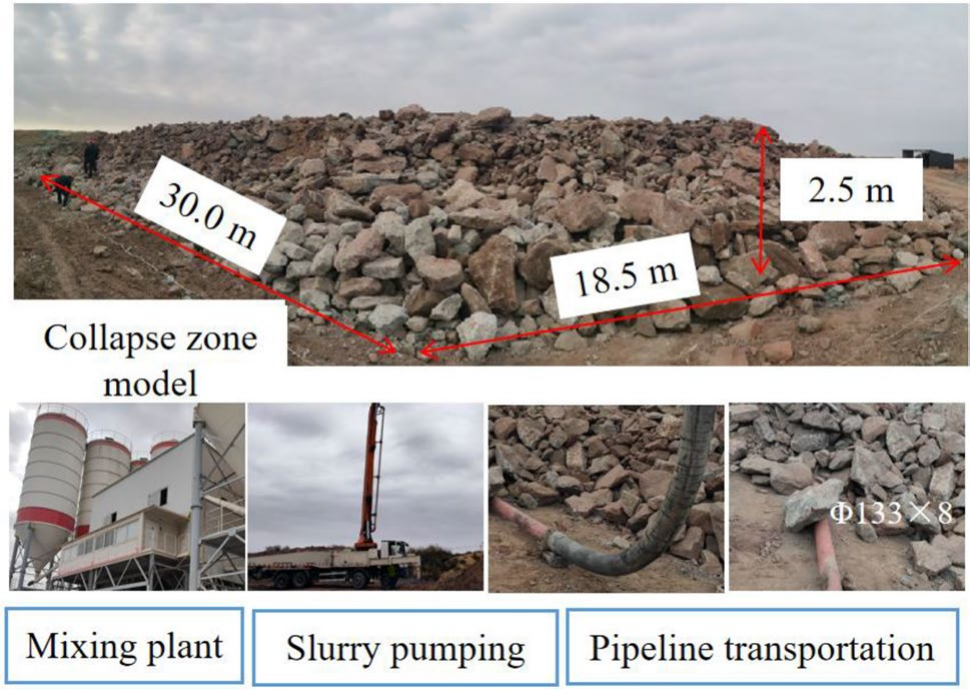
Grouting on-site process.

In order to study the fillability of gangue slurry and explore the relationship between “gangue slurry and goaf gap,” field simulation tests of grouting filling were carried out. The study area focused on the lower collapse zone of an arc-shaped triangular area, where collapsed rock accumulation displayed a step-like pattern. Due to factors such as the height of low-level grouting filling, the difficulty of modeling accumulation, and the safety and effectiveness of stacking, the researchers designed the lower collapse zone as a stepped collapse zone. Approximately 30 m behind the working face, a research area with a dip length of about 18.5 m within the dip range of the arc-shaped triangular area was selected. The strike length of this research area is 30.0 m. The first step height is approximately 0.75 m, and the second step height is approximately 2.5 m. In this way, a scaled-down representation of the collapsed rock accumulation in the lower collapse zone of the arc-shaped triangular area was approximately reconstructed on the ground with a 1:1 ratio. To consider the position relationship between the free accumulation area and the load-affected area after the mining of the working face, the model conducted partition disposal by designing different accumulation heights. The stacking height of the free stacking area (lower bench) was set at 0.75 m, while the stacking height of the load-affected area (upper bench) was set at 2.5 m. The dip section area was 30.24 m^2^, and the total volume was 1088 m^3^. To conduct the grouting filling, the slurry-filled pipeline was laid on the bottom of the model. The two boundaries of the test strike and the dip boundary were constrained by loess accumulation. This experiment aimed to observe and analyze the behavior of the gangue slurry filling in the stepped collapse zone and investigate its interaction with the goaf gap, providing valuable insights for the application of grouting filling in actual mining scenarios. The grouting site is shown in Fig. 7.

### Determination of slurry preparation param

**Figure 8:**
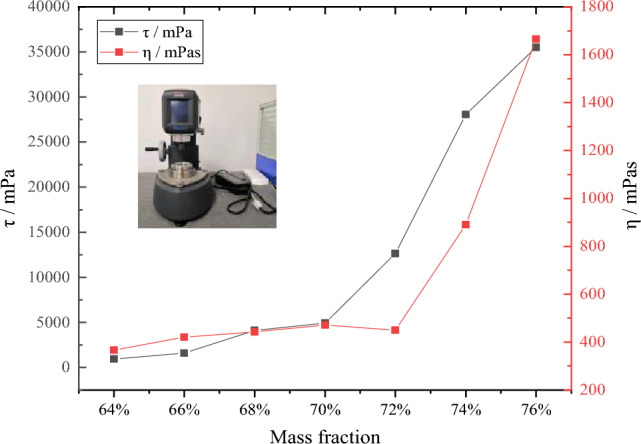
Viscosity and slurry concentration curve.

1. Slump test
  For this experiment, gangue samples with a particle size of − 200 mm were taken. Before the test, the gangue was subjected to two stages of coarse crushing using a jaw crusher, followed by high-precision crushing using a double-stage crusher, resulting in a particle size of − 8 mm for the powder. Considering the practical engineering conditions, the method of controlling the upper limit of particle size was employed to adjust the particle size distribution of the gangue powder. The naturally crushed powder (− 8 mm), as well as the sieved powders with particle sizes of − 5 mm, − 3 mm, and − 1.25 mm, were labeled as sample 1, sample 2, sample 3, and sample 4, respectively.
  To analyze the influence of gangue powder particle size distribution on the slurry-making effect, the four sets of gangue samples were separately mixed into slurries with concentrations of 65%, 70%, and 75%. The flowability and stability of the slurries were analyzed through slump tests.
  The experimental results show that under 65% concentration, sample 1 has a slump value of 283 mm, indicating relatively good stability and flowability of the slurry. However, the diffusion pattern of the slurry reveals the presence of large particles, and when the concentration is increased to 70% and 75%, obvious material accumulation is observed in the slump test, indicating poor stability and a tendency to settle, which hinders the formation of a stable and flowable slurry. Sample 2 shows improved results compared to sample 1, but at 75% concentration, similar settling and accumulation of coarse particles are observed due to the larger size of the gangue particles. When the upper limit of the solid material particle size is reduced to below 3 mm, samples 3 and 4 demonstrate good stability of the gangue powder slurry, maintaining homogeneity during the diffusion process, without significant central accumulation or large particles. Therefore, it can be concluded that samples 3 and 4 exhibit ideal gangue powder slurry properties.
  The slump test results indicate that when the particle size distribution of the gangue powder is within the range of natural breakage with an upper limit particle size smaller than 3 mm, stable slurry can be produced, meeting the requirements for slurry preparation in both samples 3 and 4. To ensure slurry stability and reduce the cost of gangue crushing, gangue powder with an upper limit particle size of 3 mm was used in the slurry preparation.
2. Rheological Tests of the Slurry
  Based on the determination of the particle size distribution of the gangue powder, slurry samples with mass concentrations ranging from 64 to 76% were prepared. The slurry's plastic viscosity and yield stress were tested using an Anton Paar rheometer. The results are shown in Fig. 8. The findings demonstrate that both the plastic viscosity and yield stress of the slurry increase with the increase in slurry concentration overall. However, when the slurry concentration exceeds 70% and 72%, the plastic viscosity and yield stress show a rapid increase with concentration. At low concentrations, the slurry exhibits non-Newtonian fluid characteristics, but as the concentration continues to increase, the rheological properties of the slurry are significantly affected. To ensure safe transportation of the slurry, a concentration of 70% was selected for the slurry preparation in this experiment.

### Relationship between gangue slurry and goaf void

#### Diffusion law of gangue slurry

**Figure 9:**
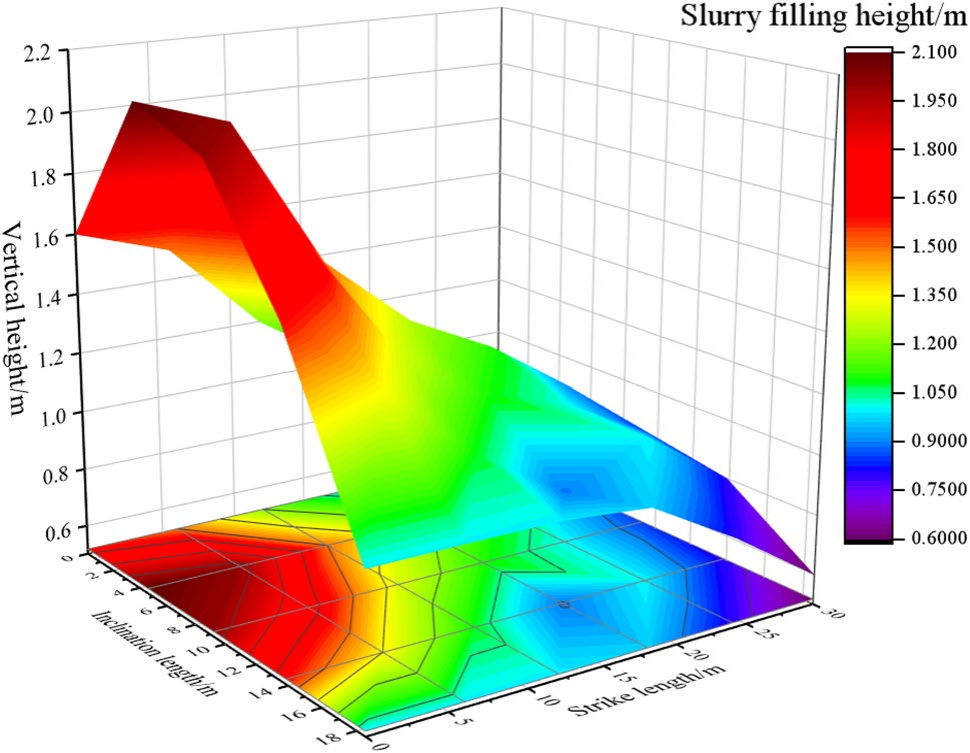
Slurry flow characteristics in goaf pores.

In order to study the relationship between the gangue slurry and the goaf gap, in combination with the size of the grouting filling site model, the measuring line is designed along the model trend direction to measure the fluidity of the gangue slurry in the goaf gap. A total of five measuring lines are arranged, which are 1.0 m, 6.0 m, 10.0 m, 18.0 m and 29.0 m away from the model boundary, and the stacked gangue is cut up along the measuring line by excavator, The flow characteristics and accumulation thickness of coal gangue slurry in the goaf gap were measured. The flow characteristics of slurry in the goaf are shown in Fig. 9.

It can be seen from Fig. 9 that the stacking height of coal gangue slurry in the goaf gap decreases with the increase of the distance from the slurry outlet, and the stacking height of slurry along the working face trend is generally greater than that along the trend. It can be seen from the analysis that the slurry pipeline is embedded on the ground, the height of the coal gangue stacked on the left side of the model is 0.75 m, and the height of the coal gangue stacked on the right side of the model is 2.5 m. Affected by the weight of the gangue, the gap on the left side is larger than the gap on the right side, so the stacking height of the slurry along the working face is generally higher than the stacking height along the trend.

With the continuous increase in slurry filling volume, when the liquid level of the gangue slurry exceeds the outflow point of the low-level grouting pipe (after the outflow period), the slurry at the spouting point in the first step of the gangue slurry flows towards the inclined boundary in a wave-like manner, causing a certain slope between the spouting point and the inclined boundary. This slope is formed during continuous low-level grouting filling and is referred to as the dynamic flow slope to distinguish it from the self-flow slope.

It can be observed that: (1) With the increase in slurry filling volume, the dynamic flow slope first decreases and then increases, and when the slurry filling volume reaches a certain capacity and continues to increase, the dynamic flow slope changes from linear to stepped. (2) When the slurry filling volume is 33 m^3^, the dynamic flow slope of the gangue slurry is 5.6%. when the slurry filling volume is 143 m^3^, the dynamic flow slope of the gangue slurry is 6.5%; when the slurry filling volume reaches 176 m^3^, the dynamic flow slope of the gangue slurry becomes stepped, with the first step of the gangue slurry having a dynamic flow slope of 7.5% and the second step having a dynamic flow slope of 1.8%.

The interaction process between coal gangue slurry and goaf voids can be divided into three stages: initial flow, vertical flow, and horizontal diffusion. In the initial flow stage, the slurry flows rapidly along the discharge port with low flow resistance due to the larger voids. With continuous grouting, the slurry flow around the discharge port is gradually restricted by the boundary loess and the retention of gangue in the free accumulation area, leading to a transition from predominantly outward flow or vertical flow to a combination of outward flow and vertical flow, accompanied by horizontal diffusion. As the slurry accumulates, the dominant flow direction shifts to horizontal diffusion, which occurs concurrently with the initial and vertical flow stages.

In the motion process of slurry material particles, the primary forces at play are “propulsive forces” and “settling forces.” “Propulsive forces” are responsible for controlling the dispersion and movement of slurry material particles in the flow direction and encompass injection pressure and shear stress. On the other hand, “settling forces” govern the dispersion and movement of slurry material particles in a direction perpendicular to the fracture surface and involve gravity and flow drag forces. For slurry material particles, the further they are from the injection point, the smaller the injection pressure they experience, and the closer they are to the fracture wall, the greater the shear stress they encounter. Consequently, slurry material particles experience lower propulsive forces at greater distances from the injection point and near the fracture wall. In contrast, the settling forces experienced by slurry material particles are solely dependent on the particle size of the particles. Larger slurry material particles experience significantly higher settling forces. The combined effect of propulsive and settling forces is particularly crucial for determining settling trajectories.

For particles of two different sizes, at the same spatial location, the difference in propulsive forces is minimal, while the difference in settling forces is substantial. This is because settling forces are influenced solely by particle size, resulting in larger particle size slurry material particles experiencing considerably higher settling forces than their smaller counterparts. As a result, the combined force of propulsive and settling forces is greater for larger particle size slurry material particles, leading to faster settling rates for larger particles. If slurry material particles are given the same initial horizontal velocity, their settling curves can be approximated as trajectories of projectile motion. Due to differences in particle size distribution, the settling trajectories for particles of various sizes vary. If the settling trajectories are plotted using the largest and smallest particle sizes as references, the trajectories for other particle sizes should fall within the range defined by these two.

In conglomerate deposits, void spaces exhibit diverse shapes and random distribution. When smaller voids appear in local areas, the flow of conglomerate slurry material within them undergoes frequent and substantial changes in velocity and direction. This results in a variable and less stable flow state for the slurry, ultimately leading to increased settling velocity in the vertical direction for coarser particles within the conglomerate slurry.

#### Diffusion characteristics of gangue slurry

**Figure 10:**
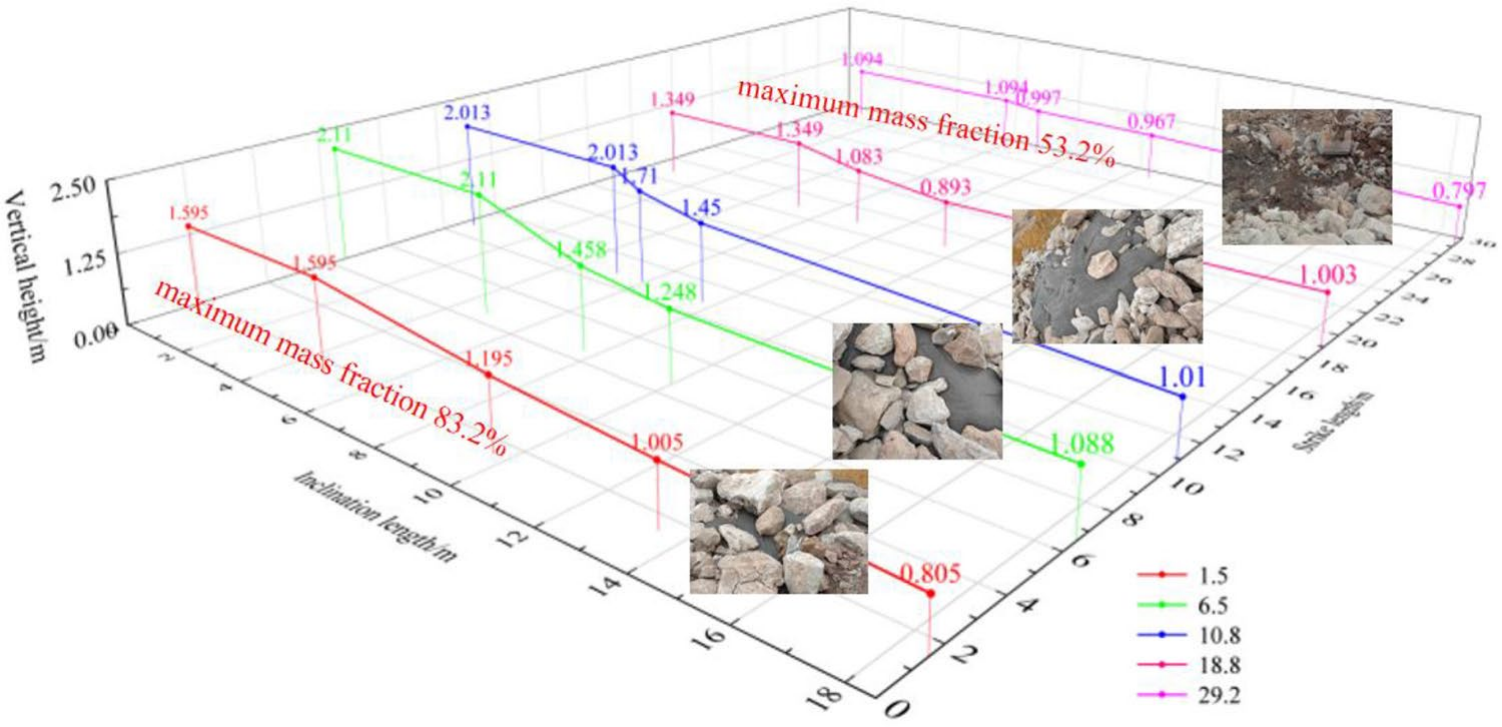
Site map of slurry flow in goaf voids.

The flow of gangue slurry in the goaf can be divided into three stages, as shown in Fig. 10. Based on on-site observations and monitoring data, it is evident that when the slurry in the first step infiltrates into the gaps between the rocks in the second step, the slurry flow exhibits the following characteristics:

The slurry is mainly distributed within the cavities formed by various rock masses and the gaps between them. Different cavities show a polarized distribution of the slurry. When the cavities are filled with slurry, the surrounding gaps between the rock masses are mostly filled with slurry, but there may still be some small gaps without slurry. On the other hand, when there is no slurry in the cavities, the slurry in the surrounding gaps between the rock masses exhibits a branched distribution, indicating that the gaps between the rock masses are clogged by slurry (possibly due to the presence of larger particles in the slurry or the gaps being too small). This indicates that the flow of slurry within the gaps in the second step is selective. According to the measurement data, the minimum width of the gaps where the slurry enters is about 7 mm.

Based on the distribution of the slurry in different parts of the cavities and gaps, it is evident that the flow of slurry in the gaps in the second step appears to be branched. In other words, the slurry flows into the cavities along the gaps between the rock masses. Once the cavities are filled, the slurry continues to flow into adjacent cavities along the remaining gaps between the rock masses. This process continues, branching out like a tree, sequentially filling voids and gaps, thereby causing the slurry to diffuse and flow deeper into the goaf.

It can be observed that the tailings slurry exhibits a selective flow pattern in the goaf. The slurry discharged from the outlet flows primarily through seepage along the dynamic flow slope in the first step. Subsequently, the slurry in the first step infiltrates the second step along the impregnation direction. The slurry flowing from the first step to the second step accounts for more than 37.8% of the total slurry. In the second step, the slurry branches out and fills the cavities and gaps between the rock masses. Accumulation points of the slurry are formed at the boundaries of both the inclination and direction, with the largest accumulation occurring at the intersection of the inclined and directional boundaries.

In the grouting filling process of the simulated collapsed zone using the low-level grouting method, significant variations were observed in the composition of the gangue slurry due to the effects of flow and deposition in different regions of the goaf. Along the strike direction, as one moves away from the low-level grouting pipe outlet, the gangue slurry exhibits a reduction in coarse particles and an increase in fine particles. Consequently, the mass fraction of the gangue slurry decreases, with the highest value being 83.2% and the lowest being 53.2%. This phenomenon can be attributed to the rapid sedimentation of coarse particles during flow and deposition, leading to their accumulation near the low-level grouting pipe outlet. Conversely, fine particles, being lighter, are more easily transported to the boundary regions due to elutriation. In contrast, in the dip direction, as one penetrates deeper into the goaf, there is a more pronounced decrease in coarse particles and an increase in fine particles compared to the strike direction. This selective influence of the goaf on the gangue slurry flow allows fine particles to effectively pass through the gaps between rock blocks and penetrate deeper into the goaf. Regarding the vertical direction, as the height increases, the proportion of coarse and fine particles in the gangue slurry remains relatively stable, but the mass fraction decreases. This decrease in mass fraction is a result of the seepage process, which causes the analysis of moisture in the gangue slurry, leading to a decrease in the shallow region. These observations underscore the significant impact of flow and deposition effects in the low-level grouting filling process on the distribution of particles within the collapsed zone. The study provides valuable insights into the behavior of gangue slurry filling in different areas of the goaf and its relationship with the original gangue slurry composition. Understanding these dynamics is crucial for optimizing grouting filling processes and ensuring efficient and effective utilization of gangue slurry in mining operations.

As the amount of gangue slurry filling increases, the dynamic flow slope initially decreases and then increases. With an increase in the gangue slurry mass fraction, the minimum width of the gaps in which gangue slurry permeates between rock blocks becomes larger, making it more challenging for the slurry to enter the deeper parts of the mined-out area. This can affect the diffusion range and filling volume of the gangue slurry within the caving zone.

The flow of gangue slurry within the mined-out area exhibits phase selectivity. The slurry that emerges from the outflow point initially flows in a seepage manner along the dynamic flow slope within the first step. Subsequently, the slurry within the first step flows in a percolation manner along the dip direction into the second step. Along the direction of gangue slurry flow, there is a decreasing trend in the proportion of coarse particles and an increasing trend in the proportion of fine particles in the gangue slurry, leading to a decrease in the gangue slurry mass fraction. By employing lower grouting filling, solid waste can be discharged into the mined-out area, and the scale of filling depends on factors such as the size of caved rock blocks, the concentration of gangue slurry, and the composition of gangue particle size.

To sum up, the action relationship between coal gangue slurry and goaf void can be divided into three stages: initial flow, vertical flow and horizontal diffusion, which are interrelated. In the initial flow stage, the slurry flows rapidly around the outlet. When it flows to a certain range, its main flow direction changes to vertical flow. The initial flow range is related to the slurry concentration, viscosity and the pore size of gangue accumulation. When the vertical flow height of slurry reaches the height of accumulation, the flow direction is mainly horizontal diffusion. It should be noted that the slurry is accompanied by a horizontal diffusion process in the process of vertical flow, and the horizontal diffusion is fast in the gangue accumulation area, and slow in the load affected area.

To enhance the effectiveness of gangue slurry backfilling, the following aspects need to be considered:

1. Simplicity, Safety, and Reliability: The system layout and process flow of slurry backfilling technology should be simple and reliable to avoid safety incidents caused by complex processes or improper arrangements, such as pipeline blockages or flooding of the working face.
2. Independence and Singular Purpose: Slurry backfilling technology aims to process solid waste, and its system layout should be independent of underground production systems. This independence ensures that it does not disrupt normal mine production and continuity plans.
3. High Efficiency and Low Energy Consumption: The processing capacity, efficiency, and cost-effectiveness of slurry backfilling technology should meet the solid waste treatment needs of modern large-scale mines while keeping energy consumption low.
4. Automation or Smart Control: Slurry backfilling technology should implement automation or smart control to reduce labor intensity, decrease the number of workers required, and lay the foundation for intelligent solid waste processing.

## Application of slurry filling engineering

### Experimental system setup

Huangling No. 2 Coal Mine is located in Huangling County, Shaanxi Province, with a production capacity of 10.0 million tons per annum, and it has two longwall working faces. For this experiment, the 301 working face in panel 3 of the mine was selected. The 301 working face has recently completed backfilling, with a relatively low degree of compaction in the goaf and weak internal water accumulation, which has minimal impact on this experiment, using a four-entry system, with two return tunnels on the south side and the main and auxiliary haulage ways on the north side.

**Figure 11:**
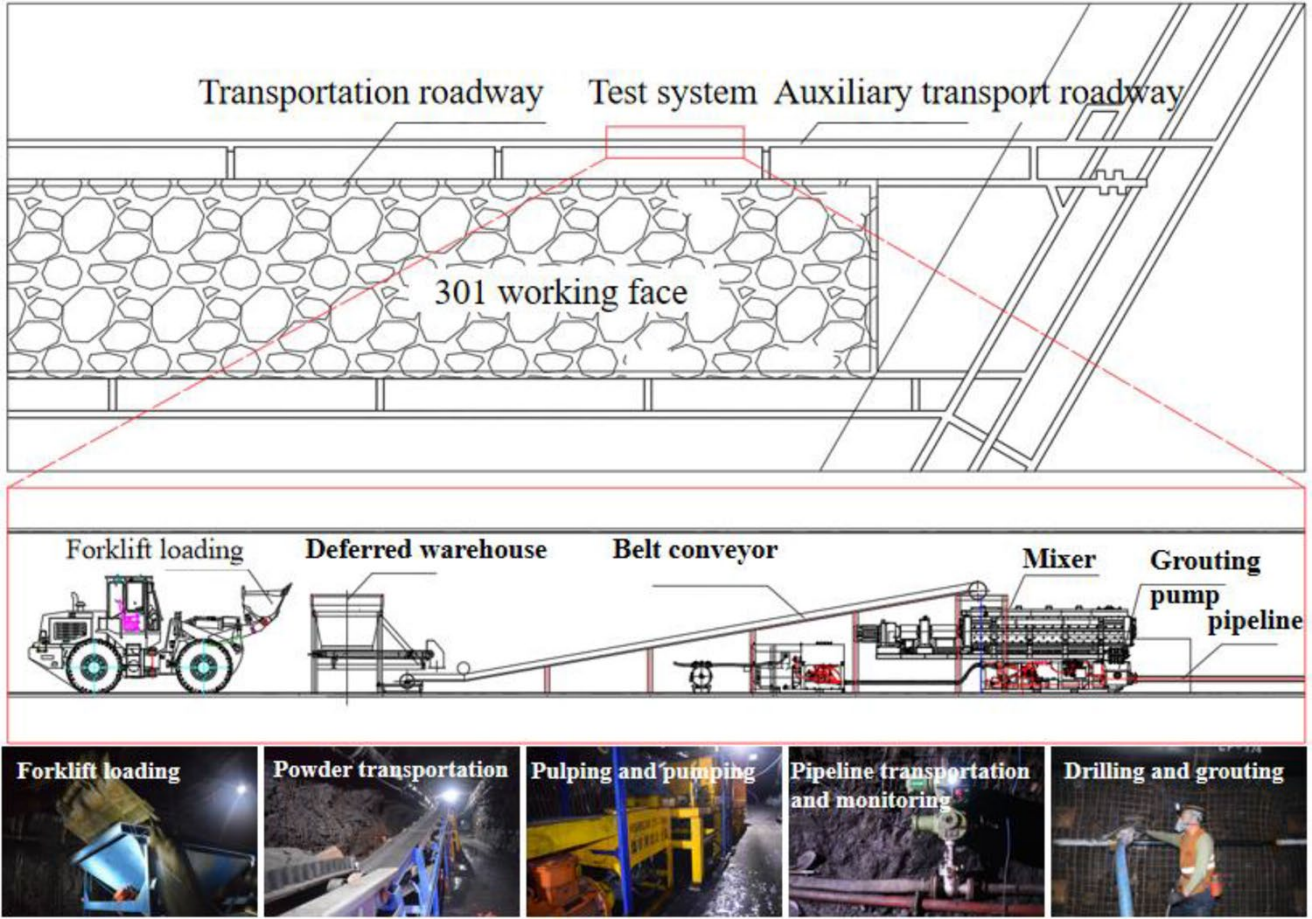
Grouting system layout.

After the completion of mining in the 301 working face, the return tunnels and main haulage way experienced roof collapse, while the auxiliary haulage way was retained for servicing the 303 working face as its return tunnel. For this experiment, the 301 working face auxiliary transport roadway of the 301 working face was selected as the test site. By installing equipment such as storage bunkers, metering feeders, belt conveyors, mixers, and grouting pumps, a complete grout filling system was constructed at the test site,as shown in Fig. 11.

The primary advantages of this technology include its surface-based system configuration, relative simplicity, minimal construction requirements, and the ability to implement integrated remote control. It also reduces labor intensity for personnel, as only slurry pipelines need to be laid underground. Furthermore, the slurry handling process is independent of coal production, resulting in minimal disruption to the mine's existing production and continuity plans.

### Grouting drilling param

The average thickness of the coal seam in the 301 working face is approximately 4.7 m, and the roof mainly consists of medium-hard sandstone. According to empirical formulas, the height of the goaf in the working face is estimated to be around 9.2 to 13.6 m. By considering the measured values of the goaf height in the adjacent 405 and 406 working faces and making analogous estimations, the goaf height in the 301 working face is calculated to be approximately 16.9 m. Therefore, the developed height of the goaf in the 301 working face is estimated to be between 9.2 and 16.9 m.

The target space for neighboring grouting is the goaf zone. In this experiment, three grouting boreholes were constructed in the auxiliary haulage way of the 301 working face at different inclinations perpendicular to the sectional coal pillar. The final depths of the three grouting boreholes were set at the top, middle, and bottom of the goaf zone, with final heights of 11 m, 6 m, and 3 m, respectively. The drilling param are listed in Table 1.

**Table 1:** Grouting drilling parameter table.

Name	Length	Elevation	Height of starting point	Height of final hole	azimuth	Casing length	Aperture	Remarks
k_1_	59.0	9.0	1.7	11.0 m	90	52.0	133	The hole shall be sealed with cement grouting, and the sealing depth shall not be less than 12 m
k_2_	42.0	5.3	1.7	6.0 m	90	41.8	133	
k_3_	40.5	1.6	1.7	3.0 m	90	37.4	133	

In this experiment, a total of 4000 tons of gangue was sampled from the surface gangue warehouse of the Huangling No. 2 Coal Mine. The gangue was crushed into gangue powder through multiple stages of crushing and screening. Before each daily experiment, 300 tons of the finished gangue powder were transported to the No. 2 auxiliary haulage way of the 301 working face using rubber-tired shuttle cars and stored temporarily. The gangue powder was then fed into the filling system using a loader, transferred by a conveyor belt into the mixer to prepare the slurry, and finally pumped into the goaf of the 301 working face through the grouting pump and slurry pipeline via the grouting boreholes.

During the experiment, grouting was conducted sequentially in the three grouting boreholes at different heights. The grouting effects, borehole inspections, and theoretical analyses were used to systematically study the feasibility of grouting in the goaf, the diffusion characteristics of the slurry in the goaf, and the arrangement of the grouting boreholes.

### Grouting test results and analysis

#### Grouting test results

**Figure 12:**
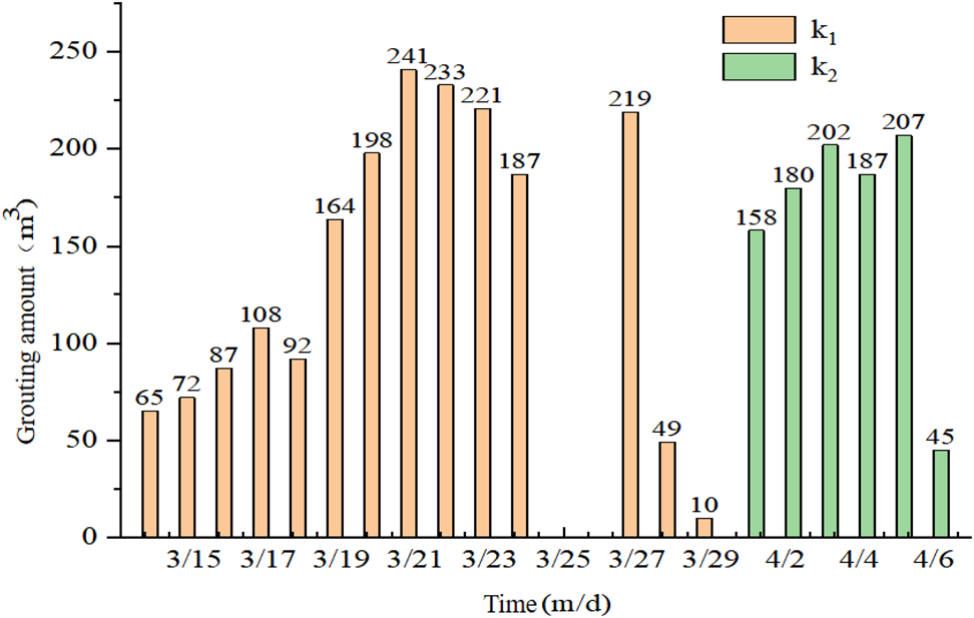
Statistics of filling volume of *k*_1_ and *k*_2_ borehole.

Based on the experimental system layout and the experimental plan, the grouting test was conducted from the innermost to the outermost regions, sequentially in *k*_1_, *k*_2_, and *k*_3_. The entire experiment lasted for 25 days. The grouting results are shown in Fig. 12.

During the grouting test, *k*_1_ borehole was grouted for a total of 16 days, with a cumulative grout volume of 1946 m^3^. *k*_2_ borehole was grouted for a total of 6 days, with a cumulative grout volume of 979 m^3^. In the initial 5 days of grouting in *k*_1_ borehole, the average daily grout volume was 84.8 m^3^/d, which was relatively small due to the operators' unfamiliarity with the process and equipment adjustments during the early stages of the test. In the mid-stage of the experiment, the grout volume reached 209 m^3^/d (with a 2 days pause), but on the 15th day, the grouting pump malfunctioned due to high oil temperature, causing the grouting to stop. The subsequent cleaning of the pipeline could not be completed, and after the equipment maintenance on the 15th day, only 10 m^3^ of grout was injected before the pressure rose too high to continue grouting, marking the end of grouting in k_1_ borehole.

For k_2_ borehole, in the first 5 days of grouting, the daily grout volume was 158 m^3^, 180 m^3^, 202 m^3^, 187 m^3^, and 207 m^3^, with an average of 186.8 m^3^/d. On the 6th day, when 45 m^3^ of grout was injected, difficulties in grouting emerged, with leakage occurring at the pipe connections and the pump pressure increasing, indicating blockages in the grouting channel. This marked the completion of grouting in *k*_2_ borehole.

As for *k*_3_ borehole, during the first attempt at grouting, it was found that the pumping pressure was higher than usual (about 14 MPa), and multiple sealing rings sequentially burst, making it impossible to proceed with grouting.

Based on the grouting test results, it can be seen that grouting in *k*_1_ and *k*_2_ boreholes was successful, with a cumulative grout volume of approximately 2925 m^3^. The practical application shows that the goaf has grouting feasibility, and the neighboring grouting filling technology has made significant breakthroughs in engineering practice.

#### Optimization of grouting process

During the course of the experiment, the grouting volume in K1 borehole was 57.8% less than the predicted value, indicating a significant deviation from expectations. An analysis of the experiment process revealed that the grouting system stopped suddenly due to a grout pump failure, and it was not possible to flush the pipeline according to the operating procedures. The operators also failed to open the valves to discharge the remaining grout in the pipeline and boreholes. As a result, a grout stoppage occurred, and when the system was repaired and grouting resumed, only 10 m^3^ of grout was successfully injected before a sharp increase in pressure and noticeable vibration indicated that the grouting channel had become blocked, preventing further grouting.

From the analysis of the grouting process, it was observed that the flow of grout in the pipeline, boreholes, and fissures was always under pressure, resulting in slow settling of the grout. However, when the grouting system suddenly stopped operating, the remaining grout in the pipeline, especially in the boreholes and fissures, would precipitate. Due to the high grout concentration, mainly composed of mudstone with certain cohesive properties, it was prone to blockages in the pipeline, especially in the boreholes and fissure channels. Therefore, during the final stages of the grouting process, it is essential to execute a water flushing procedure to clean the pipeline thoroughly. To improve system stability and ensure grouting capability, the system design should consider backup systems. In the event of an emergency system shutdown where flushing cannot be performed, it is necessary to promptly discharge the grout from the pipeline and grouting boreholes to prevent blockages.

## Conclusion

1. The formula for calculating the void ratio in the caved zone is derived, revealing the spatial distribution characteristics of the caved zone structure. The main region for backfilling is determined to be the overlapping space between the free accumulation zone and the load-influenced zone.
2. The interaction between gangue slurry and goaf voids can be divided into three stages: initial flow, vertical upwelling, and horizontal diffusion. As the amount of gangue slurry backfill increases, the dynamic slope initially decreases and then increases. Along the flow direction of the gangue slurry, a declining trend in the mass fraction of the gangue slurry.
3. The engineering application results demonstrate that the gangue slurry pipeline maintains stable transport conditions during the backfilling period, with a total grouting volume of 2925 m^3^. The flow performance of the slurry within the theoretically determined grouting area is excellent, with no occurrence of aggregation or settling.

## Data Availability

The data used to support the findings of this study are available from the corresponding author upon request.
